# Objective assessment of leg edema using ultrasonography with a gel pad

**DOI:** 10.1371/journal.pone.0182042

**Published:** 2017-08-09

**Authors:** Terumi Iuchi, Masato Kobayashi, Sayumi Tsuchiya, Naoki Ohno, Misako Dai, Masaru Matsumoto, Kazuhiro Ogai, Aya Sato, Takuto Sawazaki, Tosiaki Miyati, Shinobu Tanaka, Junko Sugama

**Affiliations:** 1 Faculty of Health Sciences, Institute of Medical, Pharmaceutical and Health Sciences, Kanazawa University, Kanazawa, Japan; 2 Wellness Promotion Science Center, Institute of Medical, Pharmaceutical and Health Sciences, Kanazawa University, Kanazawa, Japan; 3 Division of Health Sciences, Graduate School of Medical Sciences, Kanazawa University, Kanazawa, Japan; 4 Faculty of Nursing & Social Welfare Sciences, Fukui Prefecture University, Fukui, Japan; 5 Faculty of Mechanical Engineering, Institute of Science and Engineering, Kanazawa University, Kanazawa, Japan; 6 Institute for Frontier Science Initiative, Kanazawa University, Kanazawa, Japan; Instituto Butantan, BRAZIL

## Abstract

Ultrasonography (US) is useful for visual detection of edematous tissues to assess subcutaneous echogenicity. However, visualization of subcutaneous echogenicity is interpreted differently among operators because the evaluation is subjective and individual operators have unique knowledge. This study objectively assessed leg edema using US with a gel pad including fat for normalization of echogenicity in subcutaneous tissue. Five younger adults and four elderly people with leg edema were recruited. We compared assessments of US and limb circumference before and after the intervention of vibration to decrease edema in younger adults, and edema prior to going to sleep and reduced edema in the early morning in elderly people. These assessments were performed twice in elderly people by three operators and reliability, interrater differences, and bias were assessed. For US assessment, echogenicity in subcutaneous tissue was normalized to that of the gel pad by dividing the mean echogenicity of subcutaneous tissue by the mean echogenicity of the gel pad. In younger adults, the normalized subcutaneous echogenicity before the intervention was significantly higher than that after the intervention. In elderly people, echogenicity indicating edema was significantly higher than that after edema reduction. Edema was detected with accuracy rates of 76.9% in younger adults and 75.0% in elderly people. Meanwhile, limb circumference could be used to detect edema in 50.0% of healthy adults and 87.8% of elderly people. The intra-reliability was excellent (intraclass correlation coefficient > 0.9, *p* < 0.01), and the inter-reliability was good (intraclass correlation coefficient > 0.7, *p* < 0.01) for normalized subcutaneous echogenicity. Bland-Altman plots revealed that inter-rater differences and systematic bias were small. Normalized subcutaneous echogenicity with the pad can sensitively and objectively assess leg edema with high reliability. Therefore, this method has the potential to become a new gold standard for objective assessment of leg edema in clinical practice.

## Introduction

Immobility and prolonged sitting cause venous stasis and chronic leg edema in elderly people [1]. In Japan, the prevalence of edema throughout the body is 66.2% in long-term care facilities, with most edema occurring in the legs [2]. Leg edema is associated with impaired range of movement of the ankle [3], which may affect gait. Sensitive and objective assessment of leg edema results in rapid and better intervention to treat this condition.

In the clinical setting, measuring the limb circumference is the most frequently used traditional objective method [4]. However, this method indirectly assesses fluid accumulation in subcutaneous tissue because the limb includes not only subcutaneous tissue but also muscle, fibrous tissue, etc. When daily and monthly changes in subcutaneous tissue are assessed using limb circumference, a detected difference may be due to muscle atrophy, fibrous tissue deposition, and weight gain [5]. Therefore, a method to directly detect and accurately measure fluid accumulation in subcutaneous tissue is required.

Ultrasonography (US) can directly assess subcutaneous tissue and leg edema [6–8]. Edematous subcutaneous tissue is not homogeneous in US images because of the presence of water, fat, lymph vessels, etc. [9]. The echogenicity of edematous tissue is also higher than that of normal subcutaneous tissue in elderly people [6]. However, visualization of subcutaneous echogenicity is subjective because it is based on the unique knowledge of individual operators, and interpretation of US images differs with each operator [10]. For objective assessment, determining the echogenicity of edematous subcutaneous tissue normalized to the echogenicity of normal tissues (skin, muscle, and fascia) may be useful for clinical US studies. However, this technique is associated with a serious problem because the echogenicity of normal tissues in US images is unstable, especially in elderly people with chronic edema.

The aim of this study is to objectively assess leg edema in younger adults and elderly people with chronic edema using US with a gel pad for normalization of echogenicity in subcutaneous tissue. We compared the results of US assessment with the gel pad and limb circumference assessment.

## Materials and methods

### The gel pad

**Fig 1:**
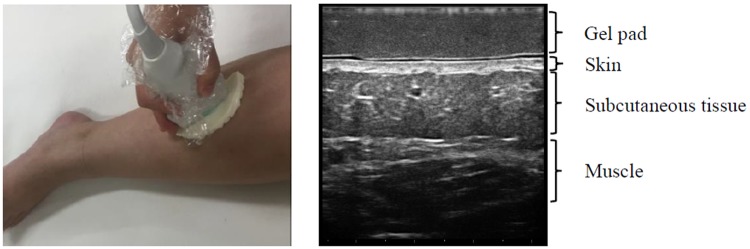
US assessments using the gel pad.

Commercially available gel pads for B-mode imaging [10] and tissue elasticity imaging [11] cannot be used for our objective assessment because these pads, which are composed mainly of water, have almost no echogenicity in B-mode. Necessary conditions for the pad were: (1) echogenicity in B-mode, (2) clear visualization of the skin, subcutaneous tissue, and muscle through the pad placed on the skin, (3) homogeneous echogenicity. We produced a new gel pad composed of 30% fat (mainly triglycerides) with an acoustic impedance of 1.46 × 10^6^ kg/m^2^s at room temperature after various examinations of the gel pad. When placed on the legs, the pad did not affect the US images of the subcutaneous tissue because the acoustic impedance values for the gel pad and water are very similar (Fig 1) [10].

### Assessment of edema in younger adults

**Fig 2:**
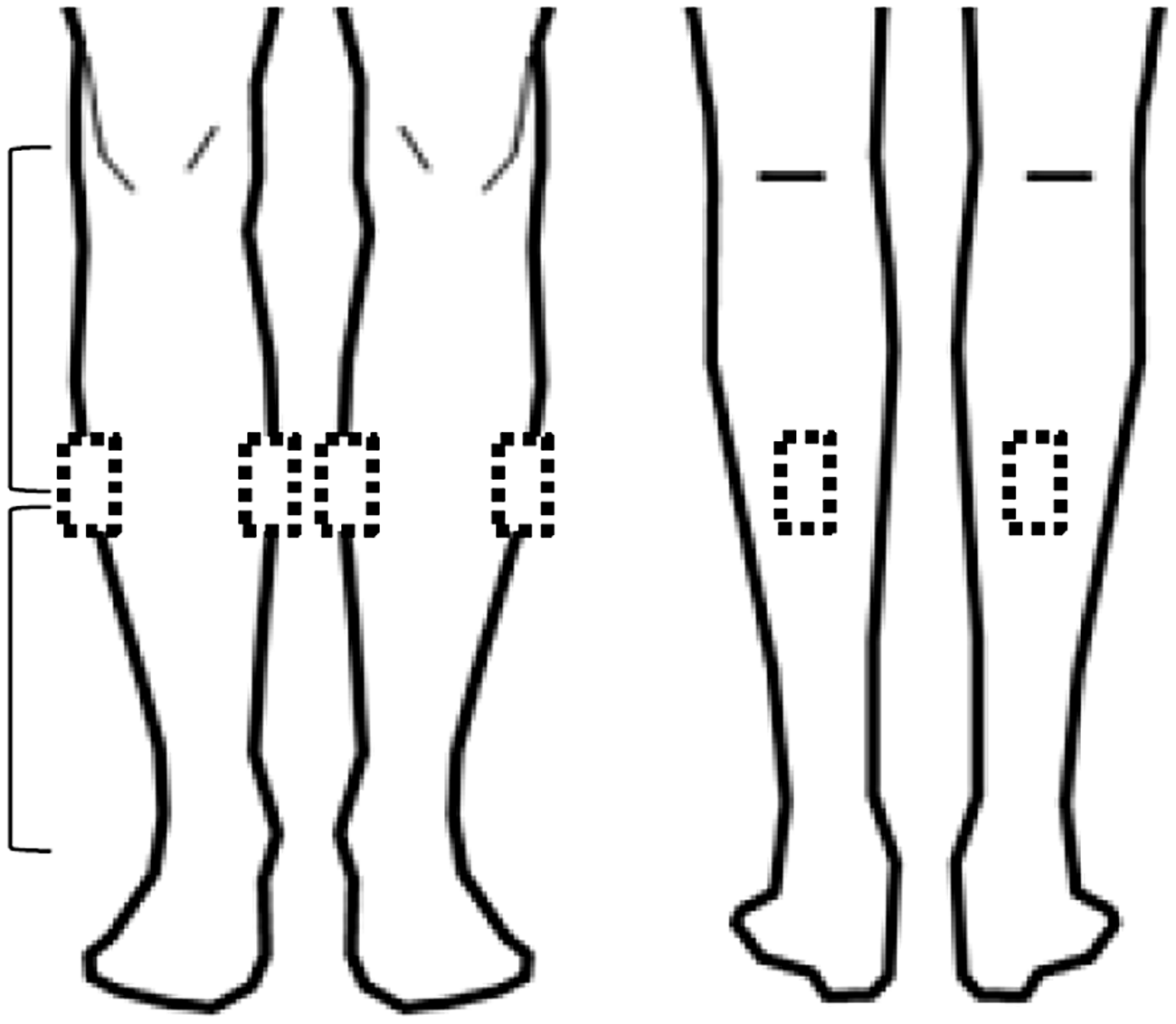
US measurement points. US measurement points of the center between the popliteal fossa and the lateral malleolus at medial, lateral, and posterior points marked with a dotted square.

The ethics committee of Kanazawa University approved this study (permit no. 509), and all participants provided written informed consent. All participants who met the following inclusion criteria were recruited: (1) age >20 years, (2) healthy women without any diseases, and (3) swollen legs after working for >8 h, primarily while sitting and/or standing. We defined edema as pitting edema. Even pressure with the right thumb was applied on the measuring points shown in Fig 2 for 10 seconds, and then the depth of pitting was assessed. Both edematous legs of five participants were assessed (total, ten legs; age range, 23 to 31 years; body mass index range, 21.2 to 23.1 kg/m^2^).

For US assessments, three operators underwent sufficient training to learn maintenance of the probe pressure. During the training, 10-mm thickness and homogeneous echogenicity of the pad were checked in US images. In addition, the probe pressure was regulated to clearly obtain US images of the lower limb subcutaneous tissue. Assessments of US and limb circumference were performed before and after a 20-min intervention of leg elevation and vibration to reduce edema [12, 13]. For vibration, we used a Rela Wave with a frequency of 47 Hz and horizontal vibration of 1.78 m/s^2^ (Global Micronics, Kashiwa, Japan) that was placed under the legs with a cushion (80 cm long, 80 cm wide, 5 cm thick) composed of urethane and expandable beads. The participants lay in the supine position and placed both elevated legs on the center of the cushion.

We used a US device (Mylab^™^ Five; Esaote, Genoa, Italy) with an 18-MHz linear probe. The settings were standardized to gain 100% and focus 15 mm. We measured the center between the popliteal fossa and the lateral malleolus at medial, lateral, and posterior points, which were marked using dotted lines on both lower limbs with the knees bent while the participants were in the supine position (Fig 2). A total of six points were assessed twice, each at the same point before and after the vibration intervention in each participant. Although the US images of each measurement point were obtained by one of three operators, they all checked and corrected the US images during US assessment.

The mean echogenicity of each point was calculated from the results of the two assessments. To obtain normalized subcutaneous echogenicity for objective assessment of US images, regions of interest (ROIs) 10 mm wide and 1 mm thick were placed over the center of the pad and subcutaneous tissue on US images. We obtained the mean echogenicity of subcutaneous tissue in the range of 47–145, with a maximum echogenicity level of 256 to remove the echogenicity of noise, from a total of 30 assessments of five participants and the superficial fascia from the ROIs [8]. The mean echogenicity of the gel pad was also obtained from a total of 30 assessments of these five participants. The echogenicity of subcutaneous tissue was normalized using the following equation:

$$\text{Normalized subcutaneous echogenicity}=\frac{mean\;echogenicity\;in\;subcutaneous\;tissue}{mean\;echogenicity\;in\;the\;gel\;pad}$$

The mean normalized subcutaneous echogenicity of the five participants decreased after the vibration intervention, and we found that edema was reduced as measured by the normalized subcutaneous echogenicity of US. On the other hand, the limb circumference at the same positions as US assessments of both lower limbs was measured twice in each participant and was used to calculate the mean unilateral circumference.

We analyzed the difference in the mean normalized subcutaneous echogenicity and the mean limb circumference before and after the intervention using the paired *t*-test and Wilcoxon signed-rank test after performing a normality test with SPSS v19 statistical software (IBM–SPSS, Inc. Chicago, IL, USA).

### Assessment of chronic edema in elderly people

The ethics committee of Kanazawa University approved this study (permit no. 556–1). Inclusion criteria for this study were (1) age 65 years or older, (2) chronic edema in the legs due to immobility and prolonged sitting, and (3) no treatment for leg edema in the daytime. Chronic edema was defined as pitting edema. Even pressure with the right thumb was applied to the measuring points for 10 seconds, and then the depth of pitting was assessed. Exclusion criteria were (1) poor general condition as judged by a nurse and (2) inability to obtain informed consent. Eight edematous legs of four subjects were assessed (one male and three females; age range, 69 to 85 years; body mass index range, 18.7 to 22.5 kg/m^2^). Measurements of US and limb circumference for each participant were evaluated in the early morning and prior to going to sleep on the same day. Edema is generally lower in the early morning because leg edema usually decreases during sleep, whereas edema is generally worse prior to going to sleep because legs usually swell during the day due to prolonged sitting.

We used a US device (Noblus; Hitachi Medical Corporation, Tokyo, Japan) with a 15- to 18-MHz linear probe. The settings were standardized to gain 15, dynamic range 70 dB, and focus 15 mm. The same measurement positions as used with younger adults were measured, except for the posterior position due to contracture (Fig 2). Therefore, four positions on both distal lower limbs were assessed in the early morning and prior to going to sleep. Normalization of subcutaneous echogenicity in US was calculated using the same equation as described above. On the other hand, the limb circumference was measured twice for both lower limbs at the same measurement position as used for US assessments and was used to diagnose edema in elderly people using the same method used for younger adults.

We analyzed the difference in the mean normalized subcutaneous echogenicity and mean limb circumference in the early morning and prior to going to sleep using the paired *t*-test or Wilcoxon signed-rank test after performing a normality test with SPSS v19 statistical software (IBM–SPSS, Inc.).

### Assessment of inter- and intra-rater reliability, differences, and bias

All assessments of elderly people were conducted by three trained researchers for measurement of inter- and intra-rater reliability. The US imaging operators and the personnel obtaining limb circumference measurements were adequately trained and were blinded to the results of each other’s measurements. Intraclass correlation coefficients (ICCs) were used to determine the reliability of the normalized subcutaneous echogenicity and limb circumferences. The ICC (1, 1) was used to determine intra-rater reliability. ICCs were calculated by measuring normalized subcutaneous echogenicity and limb circumference twice in elderly people. ICC (2, 1) was used to determine inter-rater reliability. ICCs were calculated from the mean normalized subcutaneous echogenicity and mean limb circumference in elderly people obtained by three operators. The data were analyzed using SPSS v19 statistical software (IBM–SPSS, Inc.). In addition, Bland-Altman plots were used to assess inter-rater differences and systematic bias in measurements of normalized subcutaneous echogenicity by the three operators.

## Results

### Assessment of edema in younger adults

**Fig 3:**
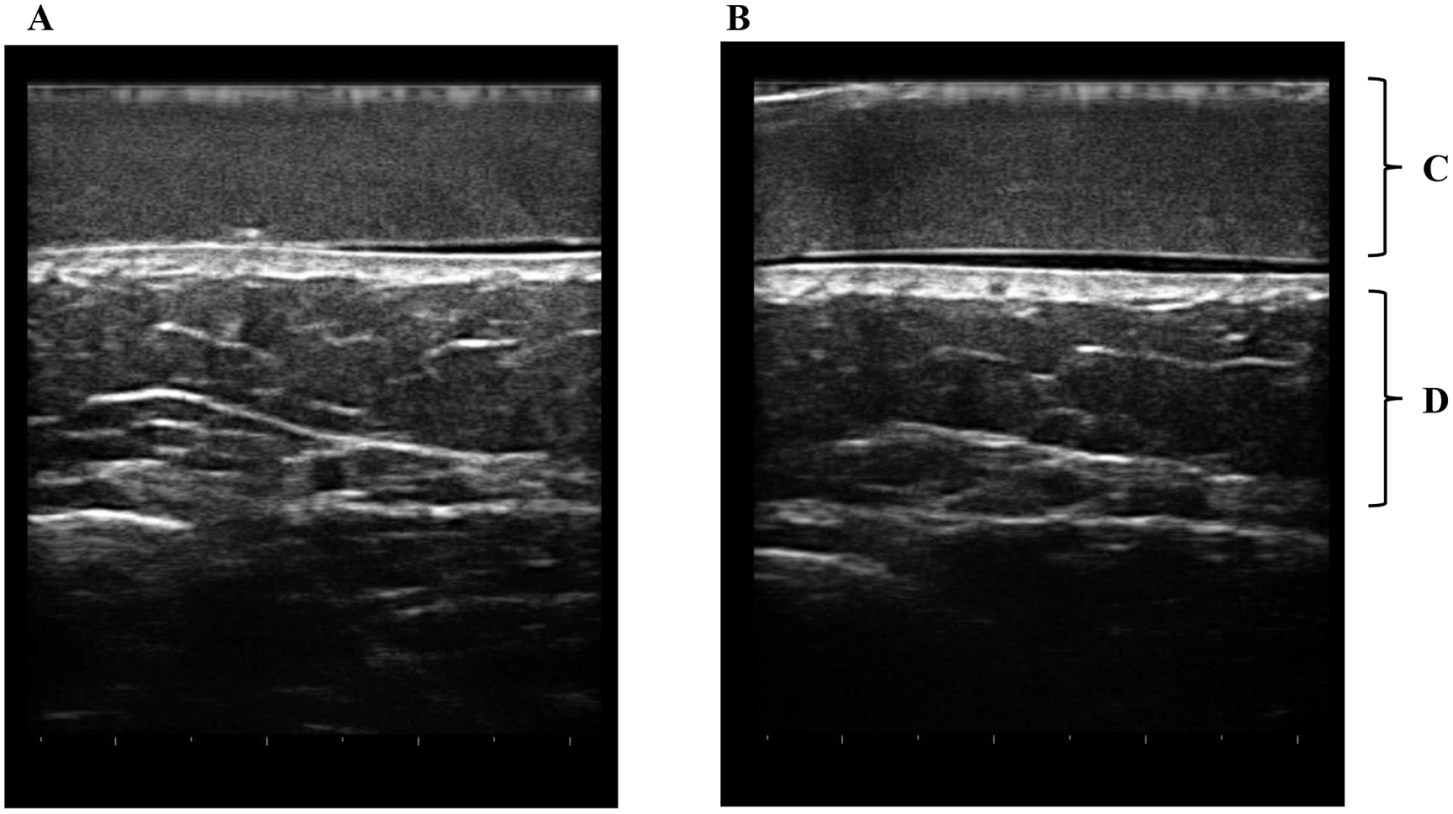
US images in a younger adult. Edema can be identified in subcutaneous tissues due to non-homogeneous echogenicity in comparison with homogeneous echogenicity of the gel pad before the intervention (A). Subcutaneous tissues become homogeneous after the intervention (B). In a younger adult, normalized subcutaneous echogenicity after the intervention (1.15 ± 0.02) was lower than that (1.21 ± 0.07) before the intervention. Mean normalized subcutaneous echogenicity (1.31 ± 0.18) in A was lower than that (1.38 ± 0.16) in B. A. Before the intervention. B. After the intervention. C. Gel pad. D. Subcutaneous tissue.

Both the gel pad and subcutaneous tissue were visualized in the same image. However, four of the 30 assessed areas were excluded; three did not clearly show subcutaneous tissue in US images, and for the other excluded area, the ROIs could not be placed over the same areas in the images before and after the intervention because the images did not match. Because the pad showed homogeneous echogenicity in US images, we could visually detect leg edema as seen by non-homogeneous echogenicity in US images (Fig 3). However, determination of whether subcutaneous tissue after the vibration intervention had visually lower echogenicity than before the intervention was difficult. Using the above formula for normalization with the pad, normalized subcutaneous echogenicity after the intervention (1.15 ± 0.02) was lower than that (1.21 ± 0.07) before the intervention in one younger adult (Fig 3). Similar results were found in 20 of 26 assessments (76.9%). In the five younger adults, the mean normalized subcutaneous echogenicity after the vibration intervention (1.31 ± 0.18) was significantly lower than that before the intervention (1.38 ± 0.16; *p* = 0.004, Table 1).

**Table 1:** The differences before and after the intervention for normalized subcutaneous echogenicity of US and limb circumference in younger adults.

	Before intervention	After intervention	*p*
Mean normalized subcutaneous echogenicity ^a^	1.38 ± 0.16	1.31 ± 0.18	0.004
Mean limb circumference ^b^ (mm)	351.1 ± 16.0	353.0 ± 16.9	0.115

^a^ n = 26, paired *t*-test.

^b^ n = 10, Wilcoxon signed-rank test.

For assessment of limb circumference, no measurements were excluded. Using all ten measurement points, the mean limb circumference was not significantly different before and after the intervention (*p* = 0.115, Table 1). Similar results were found in five of the ten measurement points (50.0%).

### Assessment of chronic edema in elderly people

**Fig 4:**
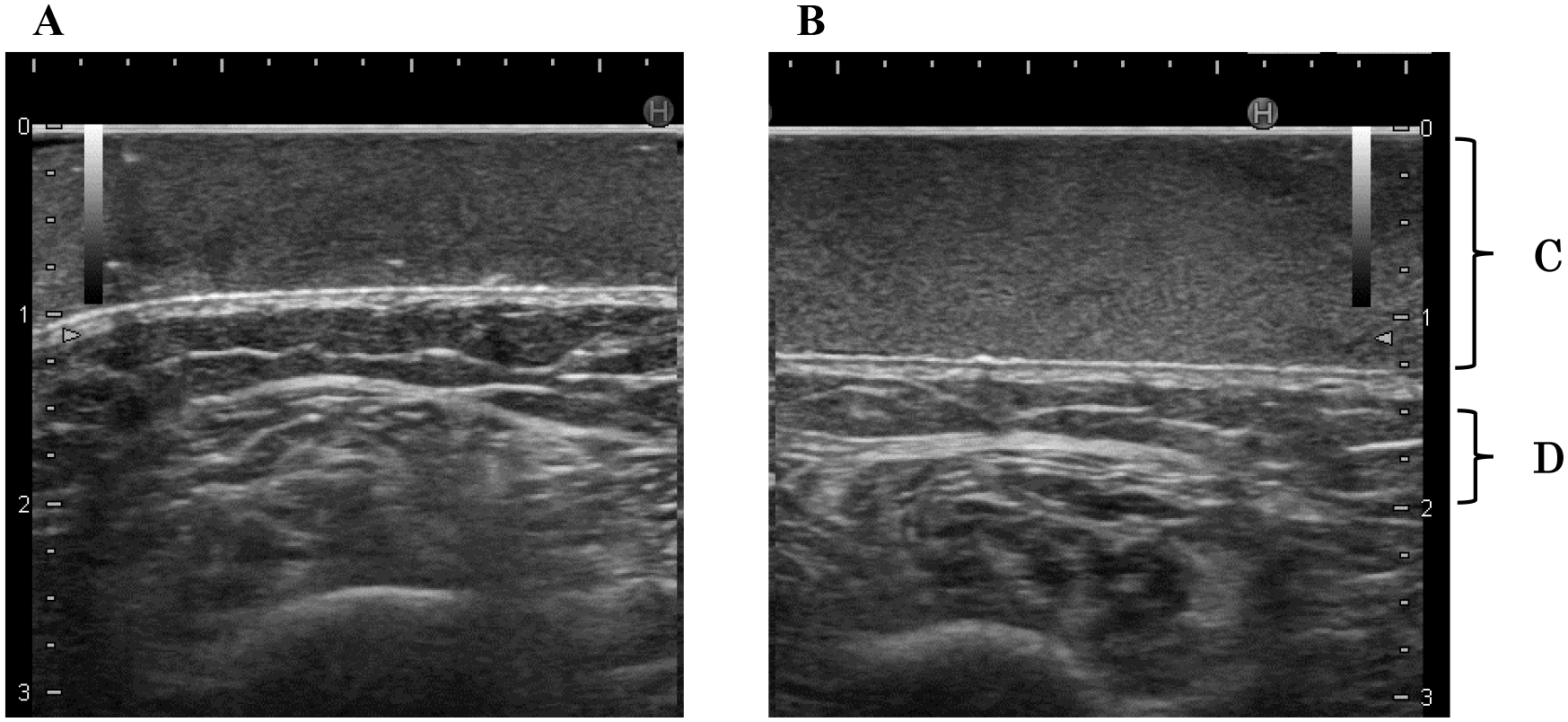
US images in an elderly individual. Chronic edema can be identified in subcutaneous tissues due to non-homogeneous echogenicity in comparison with homogeneous echogenicity of the gel pad prior to going to sleep (B). In these US images, subcutaneous echogenicity prior to going to sleep (2.60 ± 0.35) was higher than that in the early morning (1.85 ± 0.13). Subcutaneous tissues become homogeneous in the early morning (A). Mean normalized subcutaneous echogenicity (1.60 ± 0.48) in B was higher than that (1.39 ± 0.47) in A. A. Edema reduction in the early morning. B. Edema prior to going to sleep. C. Gel pad. D. Subcutaneous tissue.

For US assessment, no measurements were excluded. We could visually detect chronic leg edema compared with echogenicity of the pad because subcutaneous tissue was slightly non-homogeneous (Fig 4). In an elderly individual, subcutaneous echogenicity prior to going to sleep (2.60 ± 0.35) was higher than that in the early morning (1.85 ± 0.13) (Fig 4). The mean subcutaneous echogenicity of edema prior to going to sleep (1.60 ± 0.48) was increased in comparison with that of edema in the early morning (1.39 ± 0.47; *p* = 0.041; Table 2). Similar results were found in 12 of 16 assessments (75.0%).

**Table 2:** The differences between edema reduction in the early morning and edema prior to going to sleep for mean normalized subcutaneous echogenicity and mean limb circumference in elderly people.

	Early morning	Prior to going to sleep	*p*
Mean normalized subcutaneous echogenicity ^a^	1.39 ± 0.47	1.60 ± 0.48	0.041
Mean limb circumference ^b^ (mm)	216.0 ± 27.0	228.0 ± 27.1	0.010

^a^n = 16, paired *t*-test.

^b^n = 8, paired *t*-test.

For assessment of the limb circumference, no measurements were excluded. Using all eight measurement points, the mean circumference in legs with chronic edema was significantly higher than that when edema was reduced (*p* = 0.010; Table 2). Similar results were found in seven of eight points (87.8%).

### Assessment of inter- and intra-rater reliability, differences, and bias

For the intra- and inter-rater reliability analyses (Table 3), the ICCs (1, 1) for normalized subcutaneous echogenicity ranged from 0.923 to 0.965, and the ICC (1, 1) of the circumference was 0.999. The ICC (2, 1) for normalized subcutaneous echogenicity was 0.736, and the ICC (2, 1) for circumference was 0.998.

**Table 3:** Intra- and inter-rater reliability of normalized subcutaneous echogenicity and limb circumference in elderly people.

	Rater	Mean ± SD		ICCs (1, 1) (95% CI)	ICCs (2, 1) (95% CI)
		Rating 1	Rating 2		
Normalized Subcutaneous Echogenicity	1	1.47 ± 0.48	1.52 ± 0.50	0.923 (0.850–0.962) **	0.736 (0.559–0.864) **
	2	1.52 ± 0.42	1.48 ± 0.44	0.939 (0.879–0.962) **	
	3	1.48 ± 0.42	1.49 ± 0.39	0.965 (0.931–0.983) **	
Limb circumference (mm)	1	221.9 ± 26.8	221.8 ± 26.9	0.999 (0.998–1.000) **	0.988 (0.974–0.996)**
	2	222.6 ± 22.6	222.8 ± 27.0	0.999 (0.996–0.999) **	
	3	223.1 ± 27.4	223.3 ± 27.8	0.999 (0.998–1.000) **	

** *p* < 0.01

**Fig 5:**
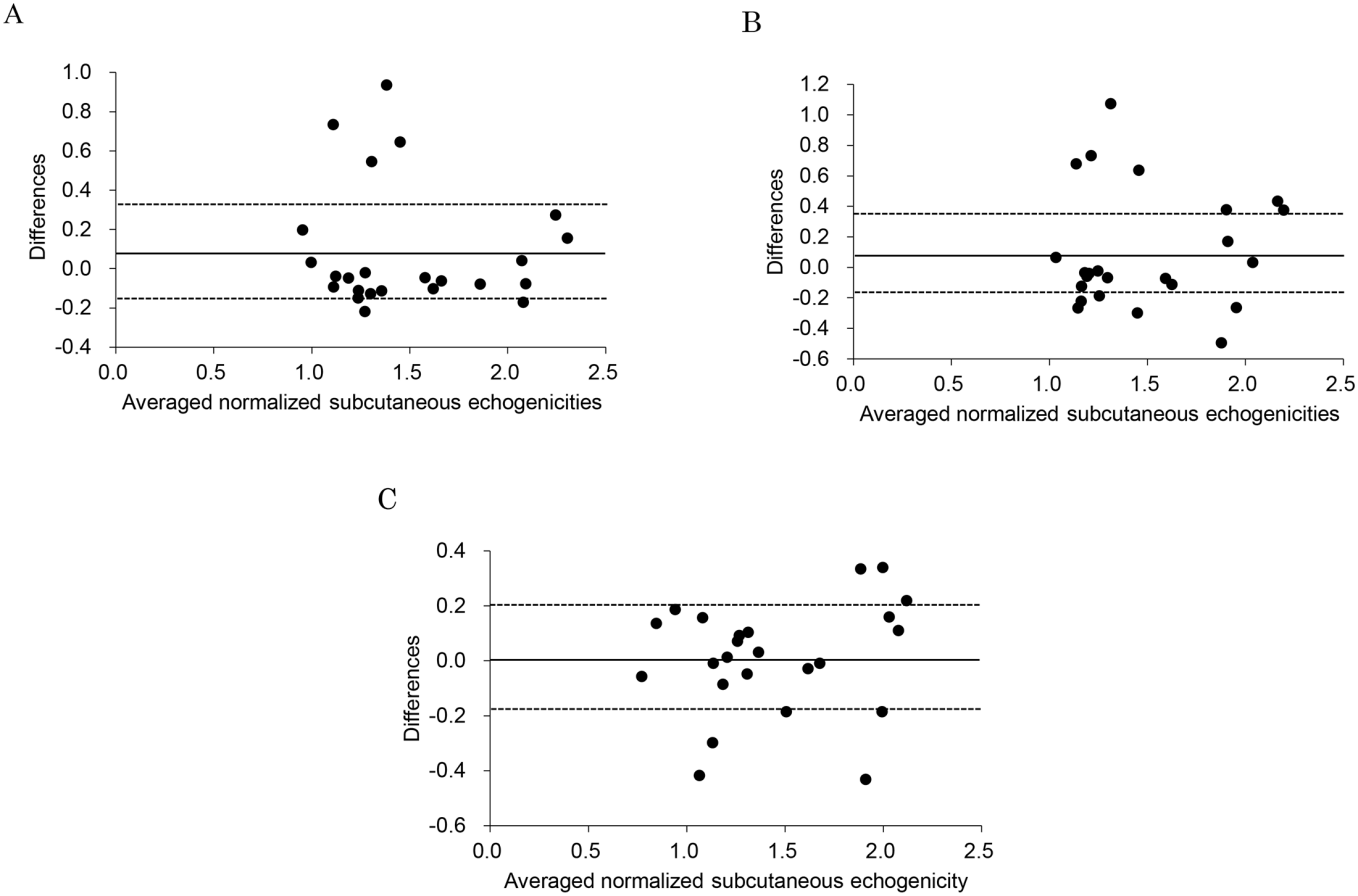
The Bland-Altman plots of normalized subcutaneous echogenicity among the three operators. Averaged differences and the 95% limits of agreement were 0.09, and -0.15 to 0.32, respectively, between operator 1 and 2 (A), 0.09, and -0.17 to 0.36, respectively, between operator 1 and 3 (B), and 0.01, and -0.18 to 0.20, respectively, between operator 2 and 3 (C). Solid and dotted lines represent the averaged differences and the 95% limits of agreement (±1.96 standard deviations), respectively.

The Bland-Altman plots in Fig 5 indicated that differences were 0.09 ± 0.31 between operator 1 and 2 (A), 0.09 ± 0.39 between operator 1 and 3 (B), and 0.01 ± 0.20 between operator 2 and 3 (C). The 95% limits of agreement were -0.15 to 0.32 between operator 1 and 2 (A), -0.17 to 0.36 between operator 1 and 3 (B), and -0.18 to 0.20 between operator 2 and 3 (C).

## Discussion

In this study, we showed that US imaging with a gel pad can be used to sensitively and objectively assess leg edema in both younger adults and elderly people. Although US has been used to assess edema in the clinical setting, subjective assessment may lead to mistakes in identification of leg edema due to different assessments by different operators. For objective assessment with US, use of the gel pad and its associated homogeneous echogenicity is helpful for visual detection of leg edema, which is non-homogeneous (Figs 3 and 4). In addition, normalized echogenicity of subcutaneous tissue with the pad can be used to objectively assess leg edema.

In younger adults, determination of whether echogenicity of subcutaneous tissue after the vibration intervention was visually lower compared to before the intervention was difficult, because the change between before and after the intervention was small. Using normalized subcutaneous echogenicity with a pad, mean normalized subcutaneous echogenicity after the vibration intervention (1.31 ± 0.18) was significantly lower than that before the intervention (1.38 ± 0.16; *p* = 0.004, Table 1). Therefore, the effect of the vibration intervention was sensitively confirmed with normalized subcutaneous echogenicity.

In elderly people with chronic edema, normalized subcutaneous echogenicity of edema prior to going to sleep (1.60 ± 0.48) was significantly higher than that following edema reduction in the early morning (1.39 ± 0.47; *p* = 0.041, Table 2). Therefore, normalized subcutaneous echogenicity can also be used to detect leg edema in elderly people with chronic edema. These results show that subcutaneous tissue has visually higher US echogenicity not only in elderly people as shown in a previous report [6] but also in younger adults. Objective assessment with US identified edema with detection rates of 76.9% in younger adults and 75.0% in elderly people in this study. On the other hand, limb circumference, which is the traditional objective assessment, detected edema in 50.0% of younger adults and 87.8% of elderly people; thus, the circumference was not significantly different before and after vibration intervention in healthy adults (Table 1). Because younger adults had less swelling compared to elderly people with chronic edema, measuring the limb circumference was not sensitive enough to detect edema in the legs of younger adults (detection rate 50.0%, five of ten measurement points). Although the detection rate of 75.0% with objective assessment of US was slightly lower than 87.8% with limb circumference in elderly people with chronic edema, both assessments have sufficient detection ability in clinical practice because we observed significant differences in both assessments when analyzing chronic edema in elderly people. Therefore, our results indicate that normalized subcutaneous US echogenicity will be a more useful technique for assessing not only leg edema in younger adults and chronic edema in elderly people, but also early stages of edema, and will be more sensitive than limb circumference measurement.

Regarding US assessment to measure leg edema, Suehiro et al. reported that US echogenicity in subcutaneous tissue increases with leg edema in immobile patients [7]. However, interpretation of US assessments differs by the individual [10]. Use of our normalized subcutaneous echogenicity for a clinical study requires confirmation of its reliability. The intra-reliability (two measurements) was excellent (ICC > 0.9, *p* < 0.01), and the inter-reliability (three raters) was good (ICC > 0.7, *p* < 0.01) for normalized subcutaneous echogenicity (Table 3). Therefore, normalized subcutaneous echogenicity showed high reproducibility. However, for measurements of limb circumference, both inter- and intra-rater reliability were excellent (ICC > 0.9), as in previous studies [14, 15]. In a previous study, water displacement and perometry methods provided excellent intra-rater and inter-rater reliabilities (ICC = 0.999 and 0.999) [15], while another study showed excellent intra-rater reliability (ICC = 0.989–1.00) for water displacement and excellent inter-rater reliability (ICC = 0.97–1.00) for perometry [16]. In this study, the intra-rater reliability was excellent for not only normalized subcutaneous echogenicity but also the limb circumference measurements, whereas the inter-rater reliability of normalized subcutaneous echogenicity was worse than that of circumference measurement and the results of previous studies that used water displacement and perometry methods. Using Bland-Altman plots, the mean differences between two operators were close to zero, and the 95% limits of agreement ranged from negative values to positive values close to zero. Therefore, although several plots exceeded the 95% limits of agreement as outliers, the systematic bias was small in the three operators. The reason may be different operation of the US probe (e.g., pressure and angle of the probe) and different measurement points assessed by each operator. Therefore, it may be necessary to provide operators with additional training to decrease the systematic bias to within the 95% limits of agreement.

US has three main advantages: (1) It is noninvasive and easy for many health professionals to perform to assess leg edema, (2) it is easy to use at the bedside (e.g., mobile handy-type US devices), and (3) it results in real-time imaging. However, objective assessment of leg edema with US has not been developed yet. By using our objective assessment of leg edema with normalized subcutaneous US echogenicity, rapid intervention and treatment of the legs may improve the patient’s well-being. Thus, normalized subcutaneous echogenicity using a pad has high potential as a new gold standard for assessment of edema in clinical practice. However, the findings of this study have limitations: (1) US operators with good skills are required to assess edema, (2) only legs and not the whole body were assessed, (3) only younger adults and elderly people were examined, (4) no obese subjects or those with severe edema, such as lymphedema, were assessed, and (5) the number of subjects in each group of younger adults and elderly people was small, because we used only one total pad for the entire experiment (about 2 hours) in each subject group due to the possibility of changes in the material in the pad over time and due to changes in the temperature of the pad itself when in contact with the skin. In the future, we must confirm if normalized subcutaneous US echogenicity assessment can be performed by less experienced US operators and if other regions of the body with edema can be detected with this method. In addition, better pads made with more stable material for US echogenicity should be developed for normalized subcutaneous US echogenicity assessment. Then, the feasibility and sensitivity percentages and ICCs should be properly investigated in a large number of subjects.

## Conclusions

Normalized subcutaneous echogenicity with a gel pad for US measurements can be used to sensitively and objectively assess leg edema with high reliability. Therefore, this method has high potential to be adapted as a new gold standard for objective assessment of leg edema in clinical practice.
